# Which Properties Allow Ligands to Open and Bind to the Transient Binding Pocket of Human Aldose Reductase?

**DOI:** 10.3390/biom11121837

**Published:** 2021-12-06

**Authors:** Anna Sandner, Khang Ngo, Christoph P. Sager, Frithjof Scheer, Michael Daude, Wibke E. Diederich, Andreas Heine, Gerhard Klebe

**Affiliations:** 1 Institut für Pharmazeutische Chemie, Philipps-Universität Marburg, Marbacher Weg 6, 35037 Marburg, Germany; anna.sandner@uni-marburg.de (A.S.); khang.ngo@online.de (K.N.); andreas.heine@staff.uni-marburg.de (A.H.); 2 Institut für Pharmazeutische Chemie, Zentrum für Tumor und Immunbiologie, Philipps-Universität Marburg, Hans-Meerwein-Straße 3, 35032 Marburg, Germany; fscheer@gmx.de (F.S.); wibke.diederich@staff.uni-marburg.de (W.E.D.); 3 Zentrum für Tumor und Immunbiologie, Core Facility Medicinal Chemistry, Philipps-Universität Marburg, Hans-Meerwein-Straße 3, 35043 Marburg, Germany; michael.daude@staff.uni-marburg.de

**Keywords:** diabetes, aldose reductase (ALR-2), binding mode, protein-ligand interaction, structure-based drug design, electronic surface potential area (ESP), transient binding pocket, specificity pocket

## Abstract

The transient specificity pocket of aldose reductase only opens in response to specific ligands. This pocket may offer an advantage for the development of novel, more selective ligands for proteins with similar topology that lack such an adaptive pocket. Our aim was to elucidate which properties allow an inhibitor to bind in the specificity pocket. A series of inhibitors that share the same parent scaffold but differ in their attached aromatic substituents were screened using ITC and X-ray crystallography for their ability to occupy the pocket. Additionally, we investigated the electrostatic potentials and charge distribution across the attached terminal aromatic groups with respect to their potential to bind to the transient pocket of the enzyme using ESP calculations. These methods allowed us to confirm the previously established hypothesis that an electron-deficient aromatic group is an important prerequisite for opening and occupying the specificity pocket. We also demonstrated from our crystal structures that a pH shift between 5 and 8 does not affect the binding position of the ligand in the specificity pocket. This allows for a comparison between thermodynamic and crystallographic data collected at different pH values.

## 1. Introduction

A large number of proteins bind substrates and endogenous inhibitors in near-surface pockets with clearly defined cavities and exhibit no major conformational differences between the *apo-* and *holo-* forms of the protein [1]. However, many proteins possess transient binding pockets which arise from functional adaptations. For example, in the case of aldose reductase (ALR-2, Enzyme Commission number (EC) 1.1.1.21), an oxidoreductase capable of processing a large scale of rather structurally diverse substrates of varying size and hydrophobicity, such a pocket only opens in the presence of particular substrate molecules. The opening creates an additional transient pocket volume of about 340 Å^3^ and involves changes in hydrophobic contacts of the pocket-flanking residue side chains along with a plane-flip of a peptide bond once an inhibitor penetrates the pocket [2]. Structural data and molecular dynamics simulations suggest that the closed conformation of the enzyme is the more stable form and at room temperature the probability of an intermediate pocket opening seems almost barred [3,4]. Yet, for binding kinetics, the opening of the pocket is not the rate-determining step of the binding process. Until now, very little was known about the thermodynamics and kinetics of the opening and closing of such transient binding pockets [4]. Nevertheless, their role in protein–protein interactions as well as in orthosteric and allosteric modulations is known to be important in many biological processes [5,6].

**Figure 1:**
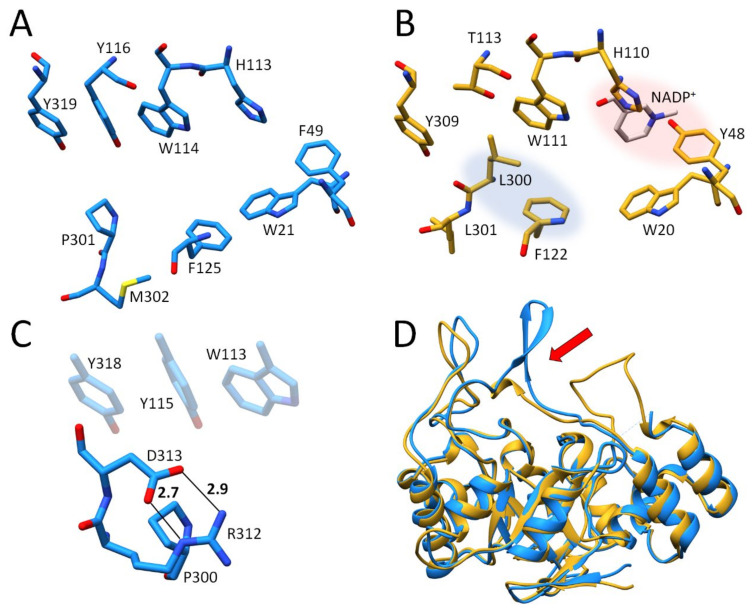
Comparison of ALR-1 and ALR-2. (**A**): Active site of ALR-1, EC 1.1.1.2 (1HQT), carbon atoms light blue. (**B**): Active site of ALR-2 EC 1.1.1.21 (this study), carbon atoms in gold. The anion binding site is highlighted in red and the specificity pocket in blue. (**C**): Salt bridges between R312 and D313 that would have to be ruptured upon a putative opening of a specificity pocket of ALR-1. (**D**): Comparison of the flexible C-terminal loops of ALR-1 and ALR-2 (highlighted with a red arrow). Superposition of ALR-1, shown as a golden ribbon, with ALR-2, shown as a blue ribbon. In this and all following figures, carbon atoms are always colored in a way to distinguish and highlight particular structures, whereas oxygen atoms are displayed in red, nitrogen atoms in blue, fluorine atoms in light green, and sulfur atoms in yellow to indicate their atom type.

Importantly for drug discovery, such a transient binding pocket may offer a selectivity advantage over proteins that have similar topology, but no such adaptive pocket. For example, this becomes apparent in the family of aldo-keto reductases. The sequence of ALR-2 matches, with 65% identity, the sequence of the closely related aldehyde reductase (ALR-1, EC 1.1.1.2) from the same protein family. The structure of the catalytic sites of these proteins is very similar, especially in the region of the rigid residues Tyr48, His110, and Trp111 (Figure 1). However, the flexible loop at the C-terminus differs significantly [7]. Due to the similar residues lining the active sites of these proteins, scaffolds used to target ALR-2 may also bind to ALR-1, causing undesired side effects [8]. Therefore, it is important to identify features of the transient pocket in ALR-2 that allow for the design of more selective and discriminating inhibitors. As mentioned, ALR-2 has a transient pocket which is not observed in ALR-1 since, in this isoform, the opening of a similar pocket would require the rupture of a strong salt bridge. Thus, inhibitors binding to the opened specificity pocket should selectively bind to ALR-2 [9]. They could consequently serve as a promising starting point for the development of novel drugs designed to treat the effects of late-stage diabetes. Such inhibitors have the potential advantage of exhibiting reduced side effects due to the inability to bind to ALR-1, which lacks an equivalent transient binding pocket.

**Figure 2:**
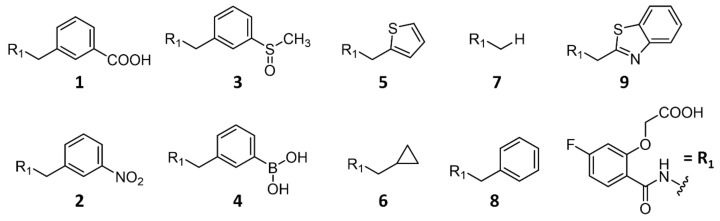
Schematic representation of ALR-2 inhibitors **1**–**9** with the parent scaffold R_1_ (lower right). These inhibitors were used to study the binding features of transient binding pocket of ALR-2. Inhibitors **1**–**2** and **7**–**9** were investigated in our previous study (Rechlin et al.) [4].

To better understand the structural and thermodynamic binding features driving the binding and accommodation in the transient pocket of ALR-2, we investigated nine inhibitors (**1**–**9**, Figure 2) in conjunction with their potential to open and occupy this pocket. Inhibitors **1** and **2** were previously reported in a study by Rechlin et al. [4] and serve as a reference in the present contribution. All nine inhibitors share a common binding motif to occupy the anion binding pocket, the previously described high affinity scaffold R_1_ (Figure 2), composed of the well-studied 2-arylcarbamoyl-phenoxy acetic acid moiety [4,10].

## 2. Results

### 2.1. Thermodynamic Data

To elucidate the binding behavior of aldose reductase inhibitors, we applied isothermal titration calorimetry (ITC). As the dissociation constants (*K*_D_) show, inhibitors **3** and **4** have single-digit micromolar affinities while **5** and **6** bind more weakly, in the two to three-digit micromolar range (Table 1). As inhibitors **5** and **6** showed low potency, the Gibbs free energy of binding (Δ*G*°) (derived from the dissociation constant (*K*_D_), determined by ITC) and the enthalpy (Δ*H*°) could not be determined with high accuracy by direct titrations. To record data at larger c-values [11,12,13,14] and thus to obtain more accurate *K*_D_ values, displacement titrations were performed with the reference inhibitor **9** (Figure S2, Supplementary Materials). Displacement titrations with the same reference, inhibitor **9**, were similarly performed for inhibitor **1**, which also showed very low affinity as previously characterized by Rechlin et al. [4]. In contrast, inhibitor **2** showed a remarkably high affinity. Since the c-value [11,12,13,14] from a direct titration of inhibitor **2** was too large and thus no reasonable *K*_D_ value could be extracted, the thermodynamic signature of this ligand was determined by a displacement titration using the weak reference inhibitor **7**.

A common phenomenon when measuring the thermodynamic parameters of a complex formation between an inhibitor and a protein is the putative superposition of changes in protonation states during protein binding [11]. Such changes can mask and thus obscure the thermodynamic signature of the binding event itself. Thus, before any reasonable analysis of the recorded thermodynamic data can be performed, such changes in the protonation states of the inhibitor and protein need to be considered and corrected. Previous measurements for the same class of ligands showed that the binding event was accompanied by an uptake of 0.8 moles of protons on average, which remains constant across the series [15]. Therefore, it is reasonable to assume that the relative difference in the thermodynamic profile remains unaffected for the whole series [15,16,17]. For this reason, all inhibitors in this series were measured solely in 10 mM HEPES (4-(2-Hydroxyethyl)-1-piperazine ethane sulfonic acid) buffer at pH 8, which allowed a relative comparison of thermodynamic binding data across the compound series. Furthermore, DMSO, added as solubility enhancer, has been described to bind to ALR-2 [18]. As weak inhibitor, DMSO will be displaced from the binding site upon accommodation of our more potent ligands. Since this contribution will likely be the same for all studied compounds, it will cancel out in our relative comparison. 

**Figure 3:**
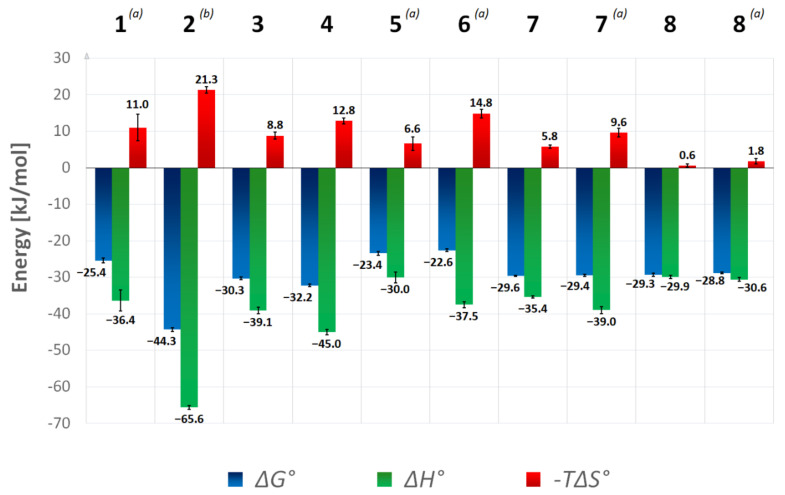
Thermodynamic parameters of ALR-2 inhibitors determined by ITC. Data for inhibitors **1**, **2**, **7**–**8** were previously collected by Rechlin et al. [4]. All measurements were performed in HEPES buffer pH 8.0 and are not corrected for overlaying protonation effects, thus only relative differences should be interpreted. Standard deviations of at least three measurements were averaged and SD’s are displayed as error bars. ^(^*^a^*^)^ Analyzed by displacement titrations using **9** as a strong inhibitor. ^(*b*)^ Analyzed by displacement titrations using **7** as a weak inhibitor.

The binding enthalpy (Δ*H*°) of the ALR-2 inhibitors was extracted from the ITC data (refer to Materials & Methods). According to the Gibbs−Helmholtz equation (Δ*G*° = Δ*H*° − *T*Δ*S*°), the entropic contribution (−*T*Δ*S*°) to binding was calculated as the difference between Δ*G*° and Δ*H*° (Table 1). Figure 3 shows the thermodynamic profiles of the inhibitors investigated, including those from our previous work for comparison [4]. 

The ITC measurements were performed by different researchers. Since different protein charges and ITC parameters, such as the ambient temperature and local humidity, may influence the ITC measurements, the comparability of the thermodynamic data collected in this work and the previously recorded data had to be assessed. To first compare the relative thermodynamic values, the high-affinity reference inhibitor **9** used for displacement titrations was measured by direct titration, and the results were compared with previous data (Table 2). Regarding the measurements of Rechlin et al., the *K*_D_ value differs by a factor of eight compared to the data measured herein. However, the thermodynamic signature of inhibitor **9** seems to be very similar considering the results of both measurements. The enthalpy contribution of the complex formation with ALR-2 is equal (−53.3 and −54.0 kJ/mol); however, a closer look reveals a difference of −*T*ΔΔ*S*° = 4.4 kJ/mol in the entropy range of the previously measured inhibitor **9**, also reflected in the affinity and Gibbs free energy [4]. In this work, we repeated the original method as closely as possible, but the measurements did not lead to identical results in the overall amount of enthalpy/entropy compensation. Thus, a direct comparison of the data from both studies should be treated with some caution. Although an absolute comparison is not possible, a relative comparison of the thermodynamic signatures of the inhibitors is, however, justified.

It has been previously demonstrated with inhibitors **1** and **2** that differences in affinity can differ significantly by exchanging a single substituent at the terminal aromatic ring. Rechlin et al. discovered that inhibitor **2**, with a nitrophenyl moiety, has a nanomolar *K*_D_, while the same scaffold decorated with an isosteric carboxylate group (inhibitor **1**) binds in the micromolar range (Table 1) [4]. Yet, the inhibitors **3** and **4** as well as **5** and **6** are similar, especially with regard to their Gibbs free energy of binding.

Although inhibitor **3** binds much less enthalpically than **4**, the sulfoxide shows a slight entropic advantage, resulting in a similar affinity of both inhibitors. The *K*_D_ values of the ITC measurements reflect the high potency of **3** and **4**, as summarized in Table 1. The same is evident for the pair **5** and **6**. Both ligands have a sole hydrophobic terminal substituent and are significantly less potent than the other inhibitors in the series. Inhibitor **5** has an entropic advantage over **6**; however, it exhibits a similar Gibbs free energy as **6** due to a less negative enthalpy value.

### 2.2. Crystal Structure Determinations

Here, eight crystal structures of complexes of wild type ALR-2 with inhibitors **1**–**8** are presented and analyzed. The obtained X-ray structures for each complex ranged from the very high resolution of 0.93 to 1.19 Å, which makes interpretation of many structural details possible (Table 3). All structures, except for the ALR-2 • **5** complex, were deposited in the PDB. The crystal structure of inhibitor **1** was previously determined by soaking at a pH of 5. In order to validate whether crystallization at pH 5 or 8 causes any impact on structure, the analysis with this ligand was repeated at the higher pH.

#### 2.2.1. Effects of Different pH Conditions on the Terminal Acetic Acid Carboxy Group at the Parent Scaffold

A comparison of inhibitor **1** in complex with ALR-2, crystallized at both pH 5 and pH 8, revealed no differences in the position of the inhibitor between the two structures (RMSD value of 0.12 Å in the position of the backbone atoms; Supplementary Materials (Figure S3)). The terminal acetic acid carboxylate group binds into the anion binding pocket in two orientations (see below) and did not differ in occupancy when crystallized at both pH values. Thus, no dependence on the pH value was observed. The p*K*_a_ value of the acetic acid moiety is below 4 [19]. However, its spatial proximity to the positively charged side chain of H110 likely supports the presence of a deprotonated carboxylate group in this region. Therefore, it can be assumed that this group of the ligand is charged in the complex at both pH 5 and 8, so a difference in the structural geometry is rather unlikely. 

#### 2.2.2. Comparison of the Binding Poses of Inhibitors 1–6

A close comparison of ALR-2 inhibitors **1**–**6** revealed that they all share a similar binding mode in the anionic binding pocket. Inhibitors **1** and **2**, investigated in our previous study [4], were used as a reference to characterize and compare the binding modes of the candidates **3**–**6** studied here.

**Figure 4:**
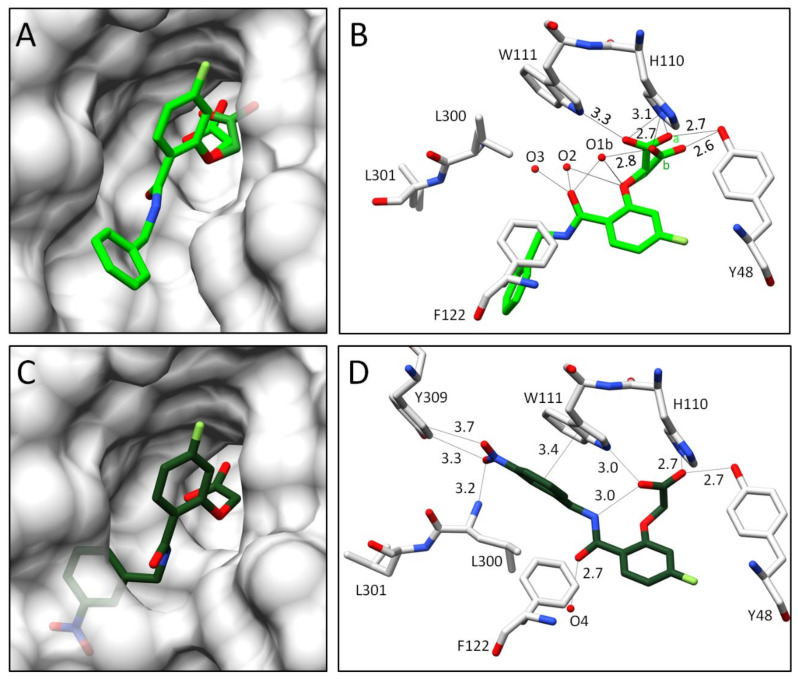
Crystal structures of inhibitors **1** and **2** in complex with wild type ALR-2. Inhibitors **1** (4YS1) [4] in light green (**A**,**B**) and **2** (4QBX) [4] in dark green (**C**,**D**) bound to the active site of ALR-2. To distinguish the position of both conformations, b is highlighted by a slightly darker color. On the left, the protein is depicted by its transparent solvent-accessible surface (light grey), whereas on the right, the interactions are indicated as black lines. Selected residues are displayed for better orientation.

With regards to scaffold R_1_ (Figure 2), which is shared by all inhibitors, the terminal carboxylate group can adopt two alternative conformations (or poses in the following). This is shown, for example, by the ALR-2 complex with inhibitor **1**, for which the structure refines to 77% inhibitor occupation (Figure 4B). In the first pose (occupancy 28%), the carboxylate group forms H-bonds simultaneously to H110, W111, and Y48, whereas in the second pose (b) (occupancy 49%) H110, Y48, and the water molecule O1b (d = 2.8 Å) are in contact. O1b interacts additionally with the inhibitor’s amide carbonyl oxygen and ether oxygen, and thereby stabilizes the bound conformation of inhibitor **1**. With this geometry, the inhibitor’s terminal benzoic acid moiety turns outward, and the specificity pocket remains in the closed state. The inhibitor protrudes from the surface of the protein (Figure 4A). The so-called gatekeeper residues, L300 and L301, adopt an ordered conformation and seal the specificity pocket. The terminal carboxylic acid function at the phenyl ring is not resolved in the F_O_ − F_C_ difference electron density at the 3*σ* level, likely due to two competitive orientations of the *meta*-attached acid group along with the enhanced residual mobility of this ligand portion. Therefore, it was not included in the final deposited structure [4]. In summary, this inhibitor binds in a well-defined conformation outside the specificity pocket, which remains in the closed state.

In comparison to inhibitor **1**, the binding pose of inhibitor **2** refines to full occupancy, as it lacks the presence of the water molecule O1b. This is accompanied by the formation of a weak, intramolecular H-bond between the inhibitor’s carboxylate and amide groups (d = 3.0 Å, Figure 4D). The latter group is flipped over by 180° and shifts the phenyl ring of the terminal group towards the former positions of the gatekeeper residues. Therefore, L300 and L301 give way and the specificity pocket fully opens to accommodate inhibitor **2** (Figure 4C). The nitro group of inhibitor **2** interacts with L300, supporting its spatial fixation in the specificity pocket. The observed non-classical secondary H-bond between the C-H dipole of the phenyl ring of Y309 and the negatively polarized oxygen atom of the nitro group has been described previously [16]. Additionally, the phenyl ring of the terminal group is stabilized by a *π-π*-stacking with the aromatic system of W111 in the opened specificity pocket (d = ~3.4 Å) [20]. In the case of inhibitor **2**, the amide carbonyl group points in the opposite direction when compared to inhibitor **1** and interacts with the water molecule O4 (d = 2.7 Å). Remarkably, the carboxylate group at the acetic acid moiety of inhibitor **2** binds with only one well-defined orientation in the anion binding pocket.

While the complex with inhibitor **1** gives rise to a closed specificity pocket, inhibitor **2** mirrors a completely opened pocket. For this reason, the poses of both inhibitors were used as reference extremes to compare the binding modes of **3**–**6** in detail. 

**Figure 5:**
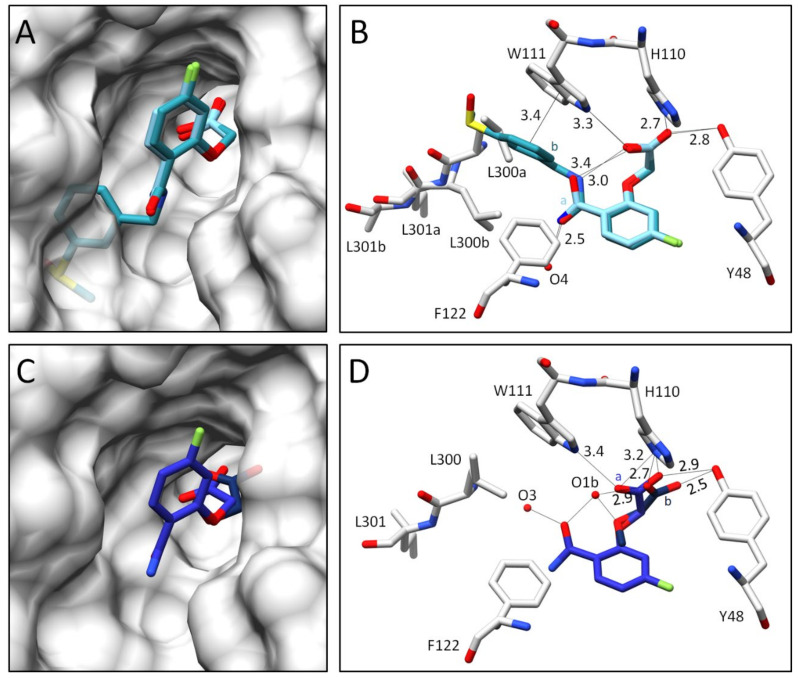
Crystal structures of inhibitors **3** and **4** in complex with wild type ALR-2. Inhibitors **3** (6TUF) in light blue (**A**,**B**) and **4** (6TUC) in blue (**C**,**D**) bound to the active site of ALR-2. To distinguish the position of the conformations, b is highlighted by a slightly darker color. On the left, the protein is depicted by its transparent solvent-accessible surface (light grey) whereas on the right, the interactions are indicated as black lines. Selected residues are displayed for better orientation.

**Figure 6:**
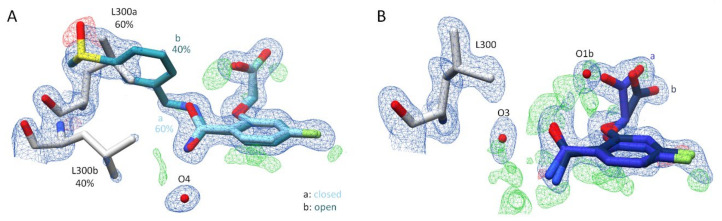
(**A**): Representation of inhibitor **3** and its electron density. Inhibitor **3** (6TUF) in light blue. To distinguish the conformations of **3**, conformation b is highlighted by a slightly darker color. Conformations a and b of the gatekeeper residue L300 are shown in grey. The respective occupancy is indicated in each case. The difference electron density map (F_O_ − F_C_) is depicted as red (negative) and green (positive) meshes at the 3*σ* level. The 2F_O_ − F_C_ density is depicted as blue mesh at the 1*σ* level. (**B**): Representation of inhibitor **4** (6TUC) in blue and its electron density. To distinguish the position of the inhibitor conformations, conformation b is highlighted by a slightly darker color. The gatekeeper residue L300 is shown in grey. The difference electron density map (F_O_ − F_C_) is depicted as red (negative) and green (positive) meshes at the 3*σ* level. The 2F_O_ − F_C_ density is depicted as blue mesh at the 1*σ* level.

Inhibitor **3** seems to bind in a complex with ALR-2 in a similar fashion to inhibitor **2**. However, a more careful examination reveals that the gatekeeper residues L300 and L301 can be refined in two different orientations (Figure 5B). Additionally, the terminal sulfoxide portion and the adjacent phenyl ring of inhibitor **3** adopt two alternative conformations, while the placement of the R_1_ scaffold adopts identical geometry for both conformations. The confirmation b of inhibitor **3** binds into the opened specificity pocket with 40% occupation. Similar to inhibitor **2**, the phenyl ring of the terminal moiety is able to stabilize the position of the inhibitor within the opened specificity pocket by *π-π*-stacking with the aromatic system of W111 (d = ~3.4 Å). However, on closer inspection of the 2F_O_ − F_C_ density (Figure 6A), L300 and L301 are also visible in an orientation that seals the specificity pocket. In this orientation, the inhibitor’s terminal portion would collide with the gatekeeper residues. The observed electron density distribution suggests that inhibitor **3** adopts a second conformation (a) and remains 60% outside of the specificity pocket, which keeps the closed state (Figure 6A). Similar to the binding pose of inhibitor **1**, any electron density indicating the placement of the terminal *meta*-attached phenylsulfoxide group in the closed state is missing. Therefore, it could not be added to the finally deposited structural model. Since the terminal portion of inhibitor **3** in conformation a is not involved in any strong directional interaction, it is likely that it remains with much higher residual mobility compared to conformation b. The split binding mode of inhibitor **3** agrees with the double conformations of the gatekeeper residues.

The boronic acid derivative inhibitor **4** refines to full occupancy and adopts an orientation outside of the specificity pocket. The gatekeeper residues keep the specificity pocket closed, which suggests that inhibitor **4** is unable to open the transient binding pocket (Figure 5D). Hence, the terminal part of the inhibitor, including the boronic acid function, is not visible in the electron density and therefore could not be modeled. Next to the anion binding pocket, a large portion of the positive difference electron density (Figure 6B) was observed, indicating that this part of the inhibitor binds with high residual mobility and likely adopts at least two alternative orientations as already described for inhibitor **1**. Furthermore, the presence of O1b next to the carboxylate group in orientation b (36% occupancy) suggests a binding mode outside the specificity pocket. 

**Figure 7:**
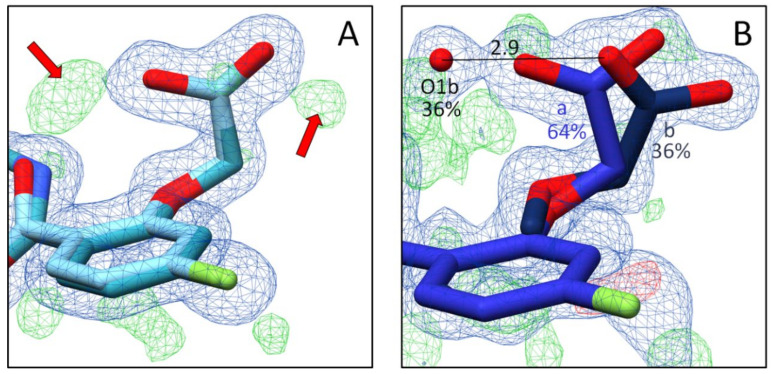
Representation of the carboxylate group of the parent scaffold of inhibitors **3** and **4**. (**A**): Inhibitor **3** (6TUF) in light blue. The red arrows indicate the likely positions of the additional water molecule O1b and the second conformation of the acetic acid moiety in the complex with **3**. (**B**): Inhibitor **4** (6TUC) in blue in complex with ALR-2. To distinguish the conformations, b is highlighted by a slightly darker color. Interactions are indicated as black lines. The difference electron density map (F_O_ − F_C_) is depicted as red and green meshes at the 3*σ* level. The 2F_O_ − F_C_ density is depicted as blue mesh at the 1*σ* level.

The binding mode of inhibitor **3** showed two orientations, either in or outside the specificity pocket. A detailed analysis of the difference electron density in the anion binding pocket (Figure 7) suggests the presence of the water molecule O1b, which agrees well with the structures of the complexes of **1** and **4**. The remaining density next to the carboxylate group can be assigned to a second placement of this group in the anion binding pocket, similar to the binding poses of the inhibitors binding outside the specificity pocket. 

Inhibitors **5** and **6** exhibit a significantly smaller terminal substituent compared to other inhibitors (Figure 2), and consequently require less space to bind to the protein. This might explain why these inhibitors deviate in their binding pose from the pattern seen for the reference inhibitors **1** and **2**. 

**Figure 8:**
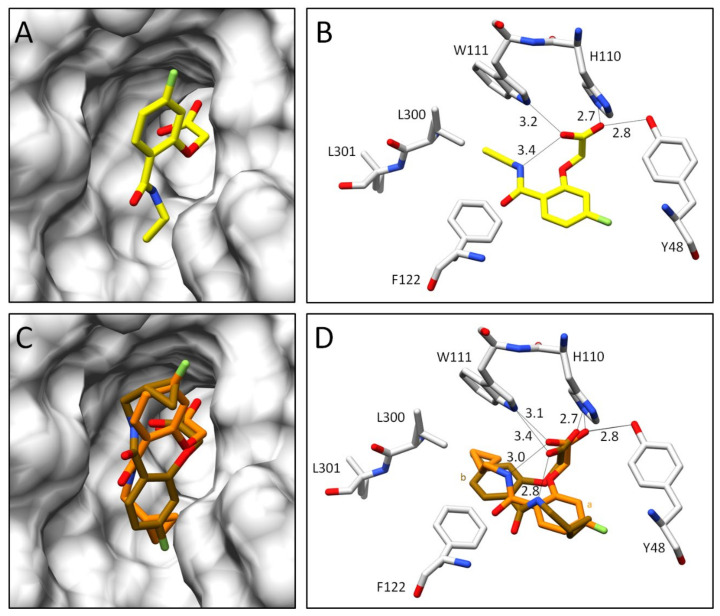
Crystal structures of inhibitors **5** and **6** in complex with ALR-2. Inhibitors **5** (not deposited) in yellow (**A**,**B**) and **6** (6SYW) in orange (**C**,**D**) bound to the active site of ALR-2. To distinguish the position of the conformations, b is highlighted by a slightly darker color. On the left, the protein is depicted by its transparent solvent-accessible surface (light grey) whereas on the right, the interactions are indicated as black lines. Selected residues are displayed for better orientation. Oxygen atoms are displayed in red, nitrogen atoms in blue, sulfur atoms in yellow, and fluorine atoms in green.

**Figure 9:**
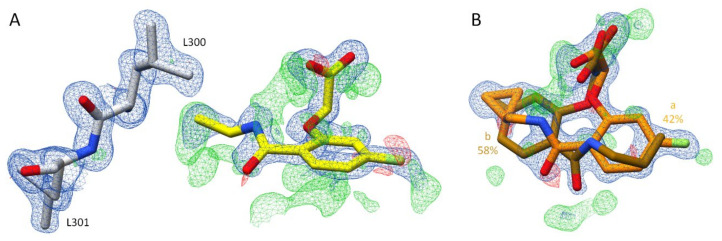
(**A**): Crystal structure of ALR-2 • **5** (not deposited) and the electron density around the ligand. Inhibitor **5** is shown in yellow; gatekeeper residues L300 and L301 are indicated in grey. Oxygen atoms are displayed in red, nitrogen atoms in blue, sulfur atoms in yellow, and fluorine atoms in green. (**B**): Crystal structure of ALR-2 • **6** (6SYW) and the corresponding electron density. Inhibitor **6** is shown in orange. To distinguish the conformations of **6**, b is highlighted by a slightly darker color. The respective occupancy is indicated in each case. The difference electron density map (F_O_ − F_C_) is depicted in both cases as red and green meshes at a contour level of 3*σ*. The 2F_O_ − F_C_ density is depicted as blue mesh at the 1*σ* level.

In the complex with inhibitor **5**, the specificity pocket remains in the closed state (Figure 8A). This is well defined by the electron density assigned to the gatekeeper residues (Figure 9A). Nevertheless, a closer inspection of the electron density next to the refined position shows that the modeled inhibitor conformation, refined to an occupancy of 87%, is not the only assignable one. However, the residual density was not sufficient to identify and model an additional conformer of inhibitor **5**. Thus, any conclusions on a split orientation of the carboxylate group in the anion binding pocket as observed for inhibitors **1** and **4**, along with the presence of the partially occupied water molecule O1b, are difficult to assign (Figure 9A). The terminal thiophene moiety, which must bind outside the specificity pocket, is not detectable in the residual density. Despite multiple data set collections for this complex and extended analysis, e.g., by generating polder maps, it was not possible to determine the position of the thiophene moiety of inhibitor **5** or to assign a second conformation of the entire inhibitor. Because of these uncertainties, the crystal structure of this complex was not deposited in the PDB. 

Additionally, in the ALR-2 in complex with inhibitor **6**, the transient specificity pocket remained closed, and no crystallographic water molecules could be assigned in the binding pocket next to the inhibitor (Figure 8C,D). As in the case of inhibitor **5**, the 2F_O_ − F_C_ electron density assigned to the gatekeeper residues undoubtedly suggests binding to the closed state (Figure 9B). However, the F_O_ − F_C_ density indicates an alternative placement of inhibitor **6** even in the closed state. In the first orientation (conformation a, 42% occupancy) the amide bond occupies a position similar to that of inhibitor **2**. This is likely possible because the cyclopropyl ring does not require much space and L300 can remain in a position usually found for the transient binding pocket in the closed state. In addition, conformation b with 58% occupation could be successfully refined (Figure 9B). Again, likely due to the minor spatial requirements of the cyclopropyl moiety, inhibitor **6** is able to flip over and bind with its fluorophenyl ring toward the gatekeeper residues. The cyclopropyl ring instead adopts the original position of the latter aromatic portion. However, with inspection of the difference electron density (F_O_ − F_C_), it can be suggested that inhibitor **6** still exhibits high residual mobility and adopts additional conformations within the binding pocket. Nonetheless, the still unexplained difference electron density does not allow modeling of further placements. The previously described inhibitor **5** has a short terminal substituent similar to **6,** and the residual difference electron density (F_O_ − F_C_) also suggests presence of further conformers. Therefore, a flipped orientation of the inhibitor may also be given in this case. Since the assigned geometry already explains 87% occupancy, no further poses were modeled for **5**.

In summary, the new inhibitors presented here, apart from inhibitor **3**, bind outside the specificity pocket, which remains in the closed state. For inhibitor **3**, both placements were observed. Binding of the terminal acetic acid moiety to the anionic binding pocket agrees well, and a split binding pose with two orientations is supposedly found in the case of binding to the closed state. The fluorophenyl ring of all inhibitors binds to the active binding site almost exclusively at the same position, except for orientation b in the ALR-2 complex with inhibitor **6**, where this ligand is flipped over. The *meta*-attached functional groups at the terminal phenyl ring (inhibitors **1**, **3**, and **4**) remain undefined in the electron density maps, likely due to residual mobility and scatter over at least two orientations. Once placed in the transient pocket, the terminal phenyl ring is well defined in the electron density (inhibitors **2** and **3**). 

## 3. Discussion

### 3.1. Structural and Thermodynamic Comparison

Previously, it was suggested that the ability of the terminal aromatic group of inhibitors **1** and **2** to penetrate and accommodate the specificity pocket does not only depend on its ability to form enthalpically favorable interactions in this pocket, but rather it also relies on the energy contribution necessary for desolvating this group upon binding [5]. In fact, to occupy the specificity pocket, either the charged carboxylate group of inhibitor **1** or the uncharged nitro group of inhibitor **2** must fully discard their hydration shell. Desolvating a charged group is by far more expansive. Thus, inhibitor **1** does not open the specificity pocket and therefore remains outside and protrudes from the protein. There, it remains partially exposed to the solvent. In contrast, the nitro group of inhibitor **2** is energetically less costly to desolvate, and a 1000-fold more potent binding into the pocket is observed. The significant decrease in the entropic contribution of inhibitor **2** compared to inhibitor **1** (Figure 3) is related to a remarkably stronger fixation of the gatekeeper residues and adjacent residues. This was concluded from a *B*-factor analysis [4]. Furthermore, the significantly higher affinity of inhibitor **2** over inhibitor **1** may additionally result from the formation of an H-bond to one of the oxygen atoms of the nitro group by the amide group L300 within the specificity pocket. A *π*-*π*-stacking of the nitrophenyl ring to the aromatic indole moiety of W111 is also partly responsible for the enthalpic advantage of inhibitor **2**. 

To validate the hypothesis that the terminal group should not bear a charged moiety, but a functional group, to undergo hydrogen bonding within the transient pocket, the sulfoxide inhibitor **3** and the boronic acid derivative inhibitor **4** were synthesized and analyzed with respect to their power to open and bind into the specificity pocket. As shown in Figure 5A–D, inhibitor **4** is unable to address the specificity pocket, whereas the sulfoxide inhibitor **3** can adopt a conformation to bind into the specificity pocket. However, it is likely that this geometry is energetically very similar to that with the terminal phenylsulfoxide outside the pocket, as this geometry is also populated in the crystal structure. 

With a p*K*_a_ value of about 9 [21], the boronic acid group of inhibitor **4** in complex with ALR-2 is protonated and thus uncharged. In that respect, it resembles inhibitor **2**. At first glance, it is surprising that the terminal boronic acid does not bind in the specificity pocket, although it would be able to form an H-bond to L300 through its hydroxyl groups, similar to the nitro moiety. The structure of inhibitor **4** in complex with ALR-2 resembles that of inhibitor **1** in terms of its binding mode and thermodynamic signature. Its improved potency results from a more favorable enthalpic contribution, possibly explained by the enthalpically less costly desolvation of the uncharged boronic acid group. 

### 3.2. Comparison of the Electronic Properties of the Terminal Aromatic Substituent

**Figure 10:**
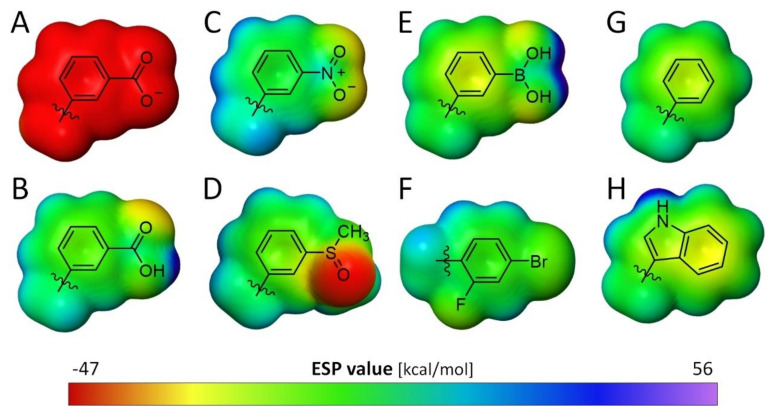
Electronic surface potential area (ESP) of the terminal aromatic inhibitor moieties and an indole ring. (**A**): benzoate moiety, (**B**): benzoic acid moiety, (**C**): nitrophenyl moiety, (**D**): phenyl sulfoxide moiety, (**E**): phenylboronic acid, (**F**): 4-bromo-2-fluoro phenyl moiety, (**G**): phenyl moiety, and (**H**): indole moiety of a tryptophan calculated with the CC-PVTZ(-F)++ basis set and the M06-2X-D3 theory level, Jaguar, Schrödinger, LLC, New York, NY, USA, 2020 [23]. The graphical representation of the potential ranges from −47 kcal/mol (red) to +56 kcal/mol (purple).

Closer inspection of the electronic properties of the preceding phenyl ring attached to the different functional groups (COOH, NO_2_, SOMe, B(OH)_2_) may provide an explanation as to why a terminal boronic acid does not match with the properties of the nitro group in inhibitor **2**, but better agrees with those of a negatively charged carboxylate group in inhibitor **1** (Figure 10). It is well known that a nitro group has a strong inductive electron-withdrawing effect. This leads to strong electron-accepting properties and transforms the adjacent phenyl ring into an electron-deficient aromatic portion [22]. Figure 10C visualizes the electronic surface potential area (ESP) of the nitro-phenyl moiety. While the substituent itself is rather electron-rich, the potential across the preceding ring is reduced (green color). 

Considering the electronic properties of the indole moiety in tryptophan, the aromatic system tends to be electron-rich due to the lack of electron-withdrawing functional groups. This is indicated by the yellow color across the ring system (Figure 10H). 

This electron enrichment of tryptophan (here W111), in combination with the electron-deficient nitro-phenyl ring of the ligand, is an ideal prerequisite for the stacking interactions of the two aromatic systems found in the complex of inhibitor **2** (d = 3.4 Å). Apart from the high desolvation costs of the carboxylate group of inhibitor **1**, the strongly enhanced charge distribution on the phenyl ring adjacent to the carboxylate group, particularly if this group is present in its deprotonated state, may prevent opening of the transient pocket along with the establishment of a stacking interaction with W111 in the complex with inhibitor **1**. 

Boronic acid, although polar and uncharged similar to the nitro group in inhibitor **2**, exerts quite different electronic effects on the adjacent phenyl ring, turning it into a rather electron-rich aromatic moiety (Figure 10D, yellow color). This charge distribution may still be detrimental for a favorable *π*-*π*-stacking with the indole moiety of W111. In consequence, the transient pocket remains sealed, and the inhibitor binds outside of the pocket. 

Steuber et al. investigated other aldose reductase inhibitors with similar scaffolds but different terminal aromatic portions [15]. There, the terminal phenyl ring was decorated by an *ortho*-fluoro and *para*-bromo substituent. Analysis of the ESP of this substituted phenyl ring (Figure 10F) reveals similar properties to a nitrophenyl moiety established by the strong electron-withdrawing effect of the attached halogen atoms (green color). Remarkably, inhibitors with this aromatic portion open the specificity pocket and bind with high potency. This supports the previously stated hypothesis that an electron-withdrawing substituent leading to an electron-deficient aromatic ring to establish a stacking geometry with the indole ring of W111 is another prerequisite for binding to the specificity pocket [10].

This hypothesis can be applied to the analog inhibitor **8**, which is able to bind into the transient specificity pocket and to interact with W111 [4]. Obviously, an unsubstituted phenyl ring is still electron-deficient enough to establish the required stacking with W111, even though inhibitor **8** is only a micromolar inhibitor of ALR-2. 

In the crystal structure the sulfoxide derivative inhibitor **3** binds at 40% to the transient pocket whereas 60% remains outside. The lack of charge on the sulfoxy group and the required electron-deficiency of the aromatic ring seem to match the necessary conditions for binding to the transient pocket. It is difficult to estimate which factor is responsible for the fact that no full occupancy of the transient pocket was observed. Factors such as the steric demand of the non-planar sulfoxy group and the non-ideal geometry of the group to interact favorably through hydrogen bonds with the pocket residues must be taken into consideration. Furthermore, it should be noted that the S = O bond is not coplanar with the phenyl ring in the adopted binding mode, breaking electronic conjugation with the π-electrons of the phenyl ring. This will definitely impact the electron-withdrawing properties of the sulfoxy group. In our calculations, coplanar geometry was assumed. As the structure of the inhibitor-**2** complex shows, the electron-withdrawing nitro group also remains in coplanar geometry within the transient binding pocket. These conformational effects of the attached groups may have an important influence on the electronic properties of the adjacent phenyl ring of the inhibitors. 

## 4. Conclusions

To determine with which conditions and energy expenditure the specificity pocket of human ALR-2 opens, two essential factors were investigated. First, the structural and thermodynamic properties of inhibitors with functional groups of different electronic nature at the terminal aromatic moiety or terminal substituents of smaller steric demand were elucidated. Second, an investigation of the electrostatic potential and charge distribution across the terminal aromatic groups of the inhibitors, and their effects on binding to the transient pocket of the enzyme, was performed.

Regarding the quantum chemical analysis of the electrostatic potential of the terminal phenyl ring of inhibitors **1**–**4** modulated by the attached substituents, the hypothesis emerged that an electron deficient aromatic group is necessary for binding into the specificity pocket [10]. In the transient pocket, an electron-rich indole moiety W111 is exposed, which creates an interaction site for *π*-stacking with the terminal aromatic group of the inhibitor. It is possible that an electron-withdrawing substituent such as a nitro group or, as stated previously by Steuber et al., an appropriate pattern of halogen substituents, can correctly adjust the electron density distribution on the terminal phenyl ring of the inhibitor. This way, the terminal aromatic substituent may undergo favorable stacking interactions with the neighboring tryptophan residue [15]. Seemingly, the unsubstituted phenyl ring in inhibitor **8** or the attachment of a sulfoxy group in inhibitor **3** generates a charge distribution just sufficient to allow stacking in the opened transient pocket. As a result, the opened and closed binding pose for inhibitor **3** are both populated in the crystal structure. Inhibitor **8** binds with its unsubstituted phenyl ring into the transient pocket, but it is equally as potent as inhibitor **7**, which lacks the terminal phenyl ring and leaves the transient pocket in closed state [4]. Thus, apart from the favorable desolvation costs of the group to be accommodated in the transient pocket, an electron-deficient terminal aromatic group supports the opening of and binding to the specificity pocket of ALR-2. It is likely that the group needs a certain volume, as smaller substituents such as a cyclopropyl group give rise to more complex binding poses with enhanced residual mobility. 

Due to the complexity of the adopted binding poses along with differences in desolvation costs and residual mobility, the correlation of differences in the thermodynamic signatures with changes in binding poses is impossible. For example, a phenyl and thiophene ring are assumed to be isosteric. Nevertheless, inhibitors **8** and **5** differ by the placement of this group. The thermodynamic profiles of both are quite different, likely also reflecting that the phenyl derivative occupies the transient pocket, whereas the thiophene analog remains outside. 

Additionally, using X-ray crystallography, we demonstrated that a shift in pH between pH 5 and 8 does not impact the binding pose of inhibitor **1** with respect to the opening of the specificity pocket. This finding is important because it proves that the crystallographic results from previous studies performed using co-crystallization are comparable to the measurements presented in this study completed by soaking. The findings also suggest that the structural data are relevant across a certain pH range. While the enzyme shows optimal enzymatic activity at a pH of 6, the value applied in previous studies during the enzyme kinetic measurements, the ITC measurements were performed at pH 8. Inhibitor soaking, on the other hand, works best with a citrate buffer at a pH of 5. To compare the data and draw conclusions across enzyme activity, thermodynamic signatures, and crystal structure, it is necessary to validate that the large pH shift between 5 and 8 does not affect the properties of the active site and thus inhibitor binding.

## 5. Materials and Methods

### 5.1. Protein Expression and Purification

Expression and purification of human aldose reductase was performed according to previously described protocols [24]. During the entire purification process, the identity and purity of the protein was constantly verified using sodium dodecyl sulfate-polyacrylamide gel electrophoresis (SDS-Page). The molecular weight of ALR-2 is about 36 kDa. Protein expression was performed using strains of *Escherichia coli* BL21 (DE3), Novagen. LB medium containing 100 µg/mL ampicillin was used as an overnight culture and was subsequently added to 1.6 L SLB medium and incubated at 310 K. Protein expression was then induced by adding 1.6 mL of 1 M isopropyl-β-d-thiogalactopyranosid (IPTG) solution. After an incubation period of 4 h, the culture was centrifuged. The harvested cells were stored at 193 K, and the supernatant was discarded. Cell disruption was performed by sonication after resuspending the pellet in buffer containing 20 mM tris(hydroxymethyl)aminomethane (Tris) and 500 mM NaCl at pH 8. The digested cell culture was applied to a chelating nickel column (HiTrap^TM^ Chelating HP, GE Healthcare; prepared with 0.1 M NiSO_4_). A brief wash step with low concentration imidazole (20 mM Tris, 500 mM NaCl, 10 mM imidazole, pH 8) was performed. A gradient up to 200 mM imidazole was used to elute the protein from the nickel column. The buffer was exchanged to 10 mM Tris pH 8.0. The tag was cleaved with thrombin (CSL Behring, Marburg, Germany) and the solution was loaded onto a HiTrap^TM^ DEAE FF Sepharose column, GE Healthcare. It was eluted through a gradient of 20 mM Tris 500 mM NaCl at pH 8.0. Purity was checked by SDS gel electrophoresis.

### 5.2. Isothermal Titration Calorimetry

Measurements were performed using an ITC_200_ (GE Healthcare) at 298 K. For the direct titrations of inhibitors **3** and **4**, the cell was filled with a 100 µM solution containing 67% active ALR-2, an excess of NADP^+^ (0.25 mM), and 3% (v/v) DMSO in 10 mM HEPES buffer at pH 8.0. For the ITC measurements, the oxidized cofactor NADP^+^ was added, which also binds to the protein but can no longer be converted. This prevents falsification of the heat signal of the inhibitor binding by chemical reactions of the cofactor, as demonstrated in previous studies [15]. The syringe was filled with the same concentration of NADP^+^ and DMSO along with inhibitor (0.75–1.0 mM) in 10 mM HEPES at pH 8.0. An initial injection of 0.3 µL, which was excluded from the analysis, was followed by 27 inhibitor injections of 1.3 µL with a duration of 0.6 s and an interval of 180 s between each injection.

For the displacement titrations of the weak inhibitors **5** and **6**, these were additionally added to the cell according to a saturation of the protein at approximately 94% and then titrated with the strong inhibitor **9**. Here, one injection of 0.3 µL was followed by 37 injections of 1.0 µL with a duration of 0.6 s and an interval of 180 s between each injection.

### 5.3. Macromolecular Crystallography

Crystallization of ALR-2 followed known protocols and was performed by the sitting drop vapor diffusion method at 291 K [9,20,24]. The protein crystallized at a concentration of 15 mg/mL in 50 mM diammonium hydrogen citrate buffer (DAHC) in conditions containing 5% (w/v) polyethylene glycol 6000 (PEG 6000), 5.2 mg/mL dithiothreitol (DTT), and 0.7 mg/mL NADP disodium salt (Carl Roth + Co. KG, Karlsruhe, Germany). An amount of 1000 µL of well solution contained 120 mM DAHC, pH 5.0 and 20% (w/v) PEG 6000. The maximum size of crystals was reached after two weeks. A total of 2 mg/mL of each inhibitor was dissolved in soaking solution (120 mM DAHC, pH 5, 25% (w/v) PEG 6000) and a crystal without visible imperfections was transferred to a 9 µL soaking droplet and fished after 24 h. The crystals were then dipped into cryoprotection buffer (120 mM DAHC, pH 5, 25% (w/v) PEG 6000). Briefly, the crystals were immersed in the cryoprotection buffer (120 mM DAHC, pH 5, 40% (w/v) PEG 6000, 1 mM inhibitor) and then frozen in liquid nitrogen.

Data collection for ALR-2 complexes **5** and **6** was performed at 100 K using BESSY II MX beamline 14.1, operated by Helmholtz-Zentrum Berlin (HZB) in Berlin, Germany. The structures of ALR-2 complexes **3** and **4** were collected at beamline 14.2 under the same conditions. The crystals belong to the P2_1_ space group, with one protein molecule in the asymmetric unit. Synchrotron radiation at preselected wavelengths and other data collection statistics are listed in Supplementary Materials (Tables S1 and S2). All data sets were indexed, processed, and scaled using XDS [25]. Molecular Replacement was performed with the CCP4 suite [26] using 4PRT as a template.

Crystallographic refinement included repeated cycles of conjugate gradient energy minimization and temperature factor refinement performed with the program Phenix [27]. Amino acid side chains were fitted to 2F_O_ − F_C_ and F_O_ − F_C_ electron density maps. The program Coot [28] was used to fit the models to the electron density maps. Data collection and refinement statistics are given in the Supplementary Materials (Tables S1 and S2). Atomic coordinates have been deposited in the PDB.

### 5.4. Ab Initio ESP Calculations

The ab initio atomic electrostatic potential charges of R1-groups of molecules **1**–**4** and **8** as well as the indole moiety of tryptophan were calculated after geometrical optimization using the density functional theory (DFT) with the M06-2X-D3 functional and the cc-pVTZ(-F)++ basis-set in the gas-phase as implemented in Jaguar 11.1 [23]. Similar calculations have been reported previously by others [29]. The M06-2X functional was selected for this study for its excellent handling of aromatic residues in terms of electrostatics and π-π stacking that are in good agreement with experimentally determined values [30]. Dispersion was mainly added for geometry optimization and the 4-bromo-2-fluoro phenyl moiety. M06-2X-D3 is viewed as one of the best hybrid density functional approximations [31,32]. The cc-pVTZ(-F)++ basis set provides good single point energies at a contained computational cost.

### 5.5. Ligand Synthesis

**Figure 11:**
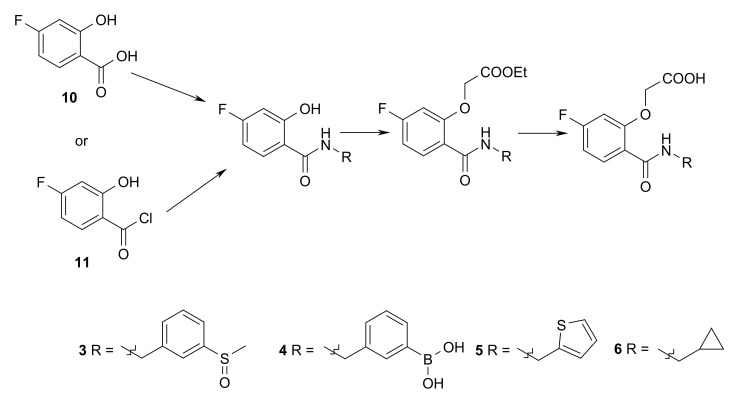
Synthesis scheme of inhibitors **3**–**6**.

The synthesis of compounds **1**, **2**, and **7**–**9** has already been described [4], and compounds **5** and **6** were synthesized accordingly utilizing 4-fluoro-2-hydroxybenzoic acid chloride **11** and cyclopropylmethanamine or thiophen-2-ylmethanamine. For the synthesis of compounds **3** and **4**, 4-fluoro-2-hydroxybenzoic acid **10** and (3-(methylsulfinyl)phenyl)methanamine hydrochloride or (3-(aminomethyl)phenyl)boronic acid were reacted utilizing HOBt and EDC*HCl as coupling reagents. The corresponding amides **12**–**15** were further reacted with ethyl bromoacetate, providing esters **16**–**19**. Cleavage of the esters under mild basic conditions rendered the desired ligands **3**–**6**, as outlined in Figure 11 [4,33]. 

The ^1^H and ^13^C NMR spectra were recorded on a JEOL ECX-400 or JEOL ECA-500 instrument. Chemical shifts (δ) are given in ppm using the residual solvent peak of DMSO-d_6_ as an internal standard (^1^H NMR: 2.50 ppm, ^13^C NMR: 39.5 ppm) [34]. Coupling constants are given in Hz, and the multiplicity of the signals is abbreviated as follows: s (singlet), d (doublet), t (triplet), q (quartet), dd (doublet of doublets), m (multiplet), and br (broad signal). NMR data refer to the signals of the main confirmation. MS spectra were recorded on a Q-Trap 2000 system with an electrospray interface (ESI). Compound purity, at least 95%, was either determined by analytical HPLC with a Shimadzu LC-10A system (reversed-phase column: Nucleodur C18, 5 μm, 100 Å, 4.6 × 250 mm, Macherey-Nagel, Düren, Germany) or by elemental analyses using a Vario MICRO cube (Elementar-Analysensysteme GmbH, Hanau, Germany). 

For synthetic intermediates, MS data are provided if available, and final compounds are fully characterized. 

**Compound 3:**

*4-fluoro-2-hydroxy-N-(3-(methylsulfinyl)benzyl)benzamide* **12**: MS (ESI+) *m/z* calculated for C_15_H_14_FNO_3_SNa [M+Na]+: 330.06; found: 330.16.

*Ethyl 2-(5-fluoro-2-((3-(methylsulfinyl)benzyl)carbamoyl)phenoxy) acetate* **16**: MS (ESI+) *m/z* calculated for C_19_H_20_FNO_5_SNa [M+Na]^+^: 416.09; found: 416.11.

2-(5-fluoro-2-((3-(methylsulfinyl)benzyl)carbamoyl)phenoxy)acetic acid **3**:

^1^H NMR (400 MHz, DMSO-*d_6_*) δ = 9.10 (t, *J* = 6.0 Hz, 1H), 7.93 (dd, *J* = 8.7, 7.1 Hz, 1H), 7.66 (s, 1H), 7.56–7.48 (m, 3H), 7.09 (dd, *J* = 11.0, 2.4 Hz, 1H), 6.96–6.90 (m, 1H), 4.93 (s, 2H), 4.61 (d, *J* = 6.1 Hz, 2H), 2.72 (s, 3H). ^13^C NMR (100 MHz, DMSO-*d_6_*) δ = 169.9, 164.3 (d, ^1^*J*_C,F_ = 248.7 Hz), 163.7, 157.1 (d, ^3^*J*_C,F_ = 10.9 Hz), 146.4, 140.8, 132.9 (d, ^3^*J*_C,F_ = 10.7 Hz), 129.4, 129.1, 122.0, 119.0, 108.1 (d, ^2^*J*_C,F_ = 21.5 Hz), 101.6 (d, ^2^*J*_C,F_ = 26.4 Hz), 65.9, 43.2, 42.5. MS (ESI-) *m/z* calculated for C_17_H_15_FNO_5_SNa [M-H]^−^: 364.06; found: 364.27.

**Compound 4: 2-(2-((3-boronobenzyl)carbamoyl)-5-fluorophenoxy) acetic acid:**

^1^H NMR (400 MHz, DMSO-*d_6_*) δ = 13.3 (s, br, 1H), 9.04 (t, *J* = 6.0 Hz, 1H), 7.96 (dd, *J* = 8.7, 7.1 Hz, 1H), 7.74 (s, 1H), 7.67–7.65 (m, 1H), 7.39–7.37 (m, 1H), 7.29–7.26 (m, 1H), 7.09 (dd, *J* = 11.0, 2.4 Hz, 1H), 6.95–6.91 (m, 1H), 4.91 (s, 2H), 4.53 (d, *J* = 6.1 Hz, 1H). ^13^C NMR (100 MHz, DMSO-*d_6_*) δ = 169.9, 164.3 (d, ^1^*J*_C,F_ = 249.5 Hz), 163.5, 157.1 (d, ^3^*J*_C,F_ = 12.0 Hz), 138.1, 134.1, 133.1, 133.0 (d, ^3^*J*_C,F_ = 10.8 Hz), 132.5, 128.9, 127.3, 119.0 (d, ^4^*J*_C,F_ = 2.4 Hz), 108.1 (d, ^2^*J*_C,F_ = 21.6 Hz), 101.7 (d, ^2^*J*_C,F_ = 26.4 Hz), 65.9, 42.9. MS (ESI+) *m/z* calculated for C_16_H_19_BFN_2_O_6_ [M+NH_4_]^+^: 365.13; found: 365.17. 

**Compound 5:**

*4-fluoro-2-hydroxy-N-(thiophen-2-ylmethyl)benzamide* **14**: MS (ESI+) *m/z* calculated for C_12_H_11_FNO_2_S: [M+H]^+^: 252.05; found: 252.05.

2-(5-fluoro-2-((thiophen-2-ylmethyl)carbamoyl)phenoxy)acetic acid **5**:

^1^H NMR (400 MHz, DMSO-*d_6_*) δ = 13.32 (s, br, 1H), 9.12 (t, *J* = 6.0 Hz, 1H), 7.96 (dd, *J* = 8.7, 7.2 Hz, 1H), 7.37 (dd, *J* = 4.9, 1.1 Hz, 1H), 7.07 (dd, *J* = 11.0, 2.3 Hz, 1H), 7.01–7.03 (sm, 1H), 6.90–6.96 (m, 2H), 4.90 (s, 2H), 4.67 (d, *J* = 6.0 Hz, 2H). ^13^C NMR (100 MHz, DMSO-*d_6_*) δ = 169.9, 164.4 (d, ^1^*J*_C,F_ = 249.5 Hz), 163.3, 157.2 (d, ^3^*J*_C,F_ = 12.0 Hz), 142.2, 133.1 (d, ^3^*J*_C,F_ = 10.8 Hz), 126.6, 125.4, 124.9, 118.7 (d, ^4^*J*_C,F_ = 2.4 Hz), 108.2 (d, ^2^*J*_C,F_ = 21.6 Hz), 101.7 (d, ^2^*J*_C,F_ = 26.4 Hz), 65.9, 37.8. MS (ESI+) *m/z* calculated for C_14_H_12_FNO_4_SNa [M+Na]^+^: 332.04: found: 332.04. Elemental analysis calculated (%) for C_14_H_12_FNO_4_S: C: 54.36, H: 3.91, N: 4.53, found C: 54.19, H: 4.10, N: 4.61.

**Compound 6:**

*N*-(cyclopropylmethyl)-4-fluoro-2-hydroxybenzamide **15**: MS (ESI+): m/z (%) = 210 (100, [M+H]^+^).

*Ethyl 2-(2-((cyclopropylmethyl)carbamoyl)-5-fluorophenoxy)acetate* **19**: MS (ESI+): *m/z* (%) = 296 (90, [M+H]^+^), 318 (60, [M+Na]^+^), 591 (100, [2M+H]^+^), 613 (55, [2M+Na]^+^). 

^1^H NMR (400 MHz, DMSO-*d_6_*) δ = 13.42 (bs, 1H), 8.67 (t, *J* = 5.3 Hz, 1H), 7.92 (dd, *J* = 8.7, 7.2 Hz, 1H), 7.08 (dd, *J* = 11.1, 2.5 Hz, 1H), 6.92 (dt, *J* = 8.6, 2.5 Hz, 1H), 4.90 (s, 2H), 3.21–3.18 (sm, 2H), 1.08–0.98 (m, 1H), 0.47–0.42 (m, 2H), 0.25–0.21 (m, 2H). ^13^C NMR (100 MHz, DMSO-*d_6_*) δ = 169.8, 164.2 (d, ^1^*J*_C,F_ = 248.3 Hz), 163.2, 156.9 (d, ^3^*J*_C,F_ = 10.8 Hz), 133.1 (d, ^3^*J*_C,F_ = 10.8 Hz), 118.8 (d, ^4^*J*_C,F_ = 2.4 Hz), 108.1 (d, ^2^*J*_C,F_ = 21.6 Hz), 101.6 (d, ^2^*J*_C,F_ = 26.4 Hz), 66.0, 43.7, 10.7, 3.4. MS (ESI+): *m/z* (%) = 268 (100, [M+H]^+^); 290 (40, [M+Na]^+^). MS (ESI+) *m/z* calculated for C_13_H_15_FNO_4_ [M+H]: 268.10; found: 268.10. Elemental analysis calculated (%) for C_13_H_14_FNO_4_: C: 58.42, H: 5.28, N: 5.40, found C: 58.12, H: 5.25, N: 5.28.

### 5.6. Associated Content

#### PDB Accession Codes

Atomic coordinates and experimental details for the crystal structures of inhibitors **1**, **2**, **7**, and **8** are available under the PDB entries 4YS1, 4QBX, 4PUU, and 4Q7B, respectively, and were published previously [4]. Crystal structures for compounds investigated herein will be released upon publication under the PDB entries listed in Table 3. Crystallographic tables can be found in the Supplementary Materials.

## Figures and Tables

**Table 1:** Thermodynamic parameters of the investigated ALR-2 inhibitor series.

Inhibitor	K_D_ [µM]	ΔG° [kJ/mol]	ΔH° [kJ/mol]	−TΔS° [kJ/mol]	Titration Mode
**1**	36.2 ± 11.4 ^(a)^	−25.4 ± 0.7 ^(a)^	−36.4 ± 2.9 ^(a)^	11.0 ± 3.6 ^(a)^	displ. ^(a,b)^
**2**	0.018 ± 0.003 ^(a)^	−44.3 ± 0.5 ^(a)^	−65.6 ± 0.5 ^(a)^	21.3 ± 0.9 ^(a)^	displ. ^(a,c)^
**3**	5.0 ± 0.6	−30.3 ± 0.3	−39.1 ± 0.9	8.8 ± 0.9	direct
**4**	2.3 ± 0.3	−32.2 ± 0.3	−45.0 ± 0.7	12.8 ± 0.8	direct
**5**	77.6 ± 15.9	−23.4 ± 0.5	−30.0 ± 1.4	6.6 ± 1.9	displ. ^(b)^
**6**	107.7 ± 16.6	−22.6 ± 0.4	−37.5 ± 0.8	14.9 ± 1.2	displ. ^(b)^
**7**	6.6 ± 0.4 ^(a)^	−29.6 ± 0.2 ^(a)^	−35.4 ± 0.3 ^(a)^	5.8 ± 0.4 ^(a)^	direct ^(a)^
**7**	7.2 ± 0.9 ^(a)^	−29.4 ± 0.3 ^(a)^	−39.0 ± 1.0 ^(a)^	9.6 ± 1.2 ^(a)^	displ. ^(a,b)^
**8**	7.5 ± 1.4 ^(a)^	−29.3 ± 0.4 ^(a)^	−29.9 ± 0.4 ^(a)^	0.6 ± 0.4 ^(a)^	direct ^(a)^
**8**	9.1 ± 0.7 ^(a)^	−28.8 ± 0.2 ^(a)^	−30.6 ± 0.5 ^(a)^	1.8 ± 0.7 ^(a)^	displ. ^(a,b)^

The thermodynamic data were determined by ITC in HEPES buffer pH 8.0. The data are not corrected for the putatively overlaying protonation effect. ^(a)^ Data were collected by Rechlin et al. [4] ^(b)^ Thermodynamic data were determined with **9** as a strong reference inhibitor. ^(c)^ Thermodynamic data were determined with **7** as a weak reference inhibitor.

**Table 2:** Thermodynamic parameters of inhibitor **9** measured in this and a previous study by Rechlin et al. [4].

Inhibitor	*K*_D_ [µM]	Δ*G*° [kJ/mol]	Δ*H*° [kJ/mol]	–*T*Δ*S*° [kJ/mol]	Titration Mode
**9** (from this study)	0.42 ± 0.08	−36.4 ± 0.5	−53.3 ± 0.5	16.9 ± 0.7	direct
**9** (derived from [4])	0.05 ± 0.01	−41.5 ± 0.4	−54.0 ± 1.0	12.5 ± 1.1	direct

The thermodynamic data were determined by ITC in HEPES buffer pH 8.0. The data are not corrected for the overlaying protonation effect.

**Table 3:** Summary of the crystallographic data of investigated ALR-2 inhibitor complexes, their PDB-codes, and the opening status of the transient specificity pocket.

Inhibitor	PDB Code	Resolution [Å]	Pocket State
**1**	4YS1 ^(a)^	1.07	closed
**2**	4QBX ^(a)^	0.98	open
**3**	6TUF	1.15	hybrid
**4**	6TUC	1.06	closed
**5**	not deposited	0.93	closed
**6**	6SWY	0.93	closed
**7**	4PUU ^(a)^	1.14	closed
**8**	4Q7B ^(a)^	1.19	open

^(a)^ Crystallographic data determined by Rechlin et al. [4].

## Data Availability

The study does not report any data.

## Supplementary Materials

The following are available online at https://www.mdpi.com/2218-273X/11/12/1837/s1. Raw ITC thermograms, crystallographic tables, supplementary references.

## Abbreviations

Å	Ångström (1 Å = 10^−10^ m)
ALR-1	Aldehyde Reductase
ALR-2	Aldose Reductase
*B*-factor	Debye-Waller factor
DAHC	diammonium hydrogen citrate
DMSO	Dimethyl sulfoxide
DTT	Dithiothreitol
EC	Enzyme Commission number
EDC	1-Ethyl-3-(3-dimethylaminopropyl)carbodiimid-hydrochlorid
ESI	Electrospray ionization
ESP	Electronic Surface Potential area
F_C_	Calculated structure amplitudes
F_O_	Observed structure amplitudes
g	Gram
*G*°	Gibbs free energy
*H*°	Enthalpy
H-bond	Hydrogen bond
HEPES	4-(2-Hydroxyethyl)-1-piperazine ethane sulfonic acid
HOBt	1-Hydroxybenzotriazol
HPLC	High Performance Liquid Chromatography
IC_50_	Half-maximum inhibitory concentration
IPTG	Isopropyl-β-d-thiogalactopyranosid
ITC	Isothermal titration calorimetry
K	Kelvin
k	Kilo
*K*_a_	Association constant
*K*_D_	Dissociation konstant
M	Molarity (mol/L)
min	Minute
MS	Mass spectroscopy
NADP^+^	Nicotinamide adenine dinucleotide phosphate
NADPH	Nicotinamide adenine dinucleotide phosphate, reduced
NMR	Nuclear magnetic resonance
PAGE	Polyacrylamide gel electrophoresis
PBS	Phosphate buffered saline
PEG	Polyethylen glycol
pH	Potentialis hydrogenii
p*K*_a_	Logarithmic acid dissociation constant
*R*-factor	Reliability factor
*S*°SD	EntropyStandard Deviation
SDS	Sodium dodecyl sulfate
T	Absolute temperature
TRIS	Tris(hydroxamethyl)aminomethane
X-ray	Röntgen radiation
