# Supplementary material for: Which Properties Allow Ligands to Open and Bind to the Transient Binding Pocket of Human Aldose Reductase?
Source: Biomolecules. 2021 Dec 6;11(12):1837. doi: 10.3390/biom11121837 (PMC8699021; doi:10.3390/biom11121837)
Supplement: Supplementary file 1 [file biomolecules-11-01837-s001.zip › biomolecules-1462491-SI.pdf]

Supporting Information

Which Properties Features Ligands to Open and Bind to the Transient Binding Pocket of Human Aldose Reductase?

Anna Sandner, Khang Ngo, Christoph P. Sager, Andreas Heine, and Gerhard Klebe*

Institut für Pharmazeutische Chemie, Philipps-Universität Marburg, Marbacher Weg 6, 35037 Marburg, Germany; anna.sandner@uni-marburg.de (A.S.); khang.ngo@uni-marburg.de (K.N.); andreas.heine@staff.uni-marburg.de (A.H.)

* Correspondence: gerhard.klebe@staff.uni-marburg.de; Tel.: +49-6421-28-21313

Received: date; Accepted: date; Published: date

Table of Contents

Figure S1: Examples of raw thermograms and integrated heat values for one of at least three direct ITC measurements of the inhibitors 3, 4 and 9……………………………………………2
Figure S2: Examples of raw thermograms and integrated heat values for one of at least three displacement ITC measurements of the inhibitors 5 – 6……………………………………….3
Figure S3: Superposition of the crystal structures of ALR-2 • **1** obtained at pH 5 and 8 ……..4
Table S1 - S2: Crystallographic Tables……………………………………….………………5
Supplementary References…………………………………………………………………..…7

Supplementary Figures

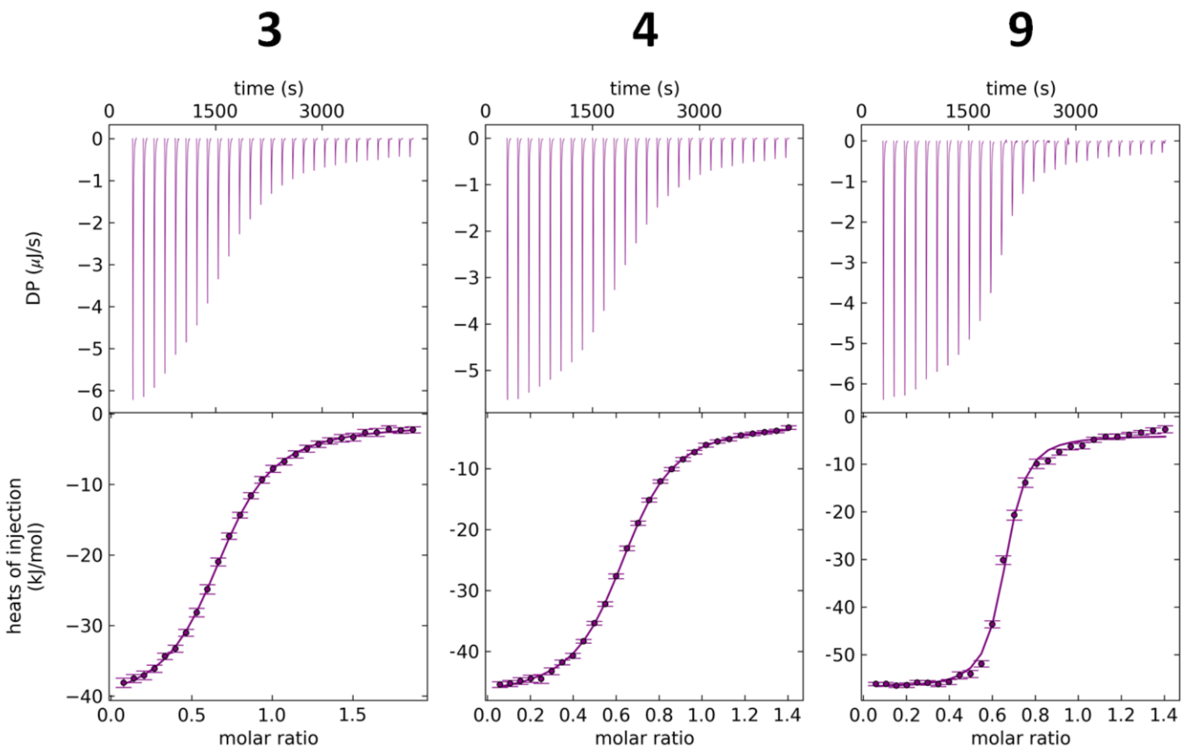

Figure S1: Examples of raw thermograms and integrated heat values for one of at least three direct ITC measurements of the inhibitors **3**, **4** and **9.** For the thermogram on the top the y-axis shows the differential power in µJ/s and the x-axis the measuring time in s. The y-axis of the evaluated data below shows the heats of injections in kJ/mol and the x-axis the molar ratio.

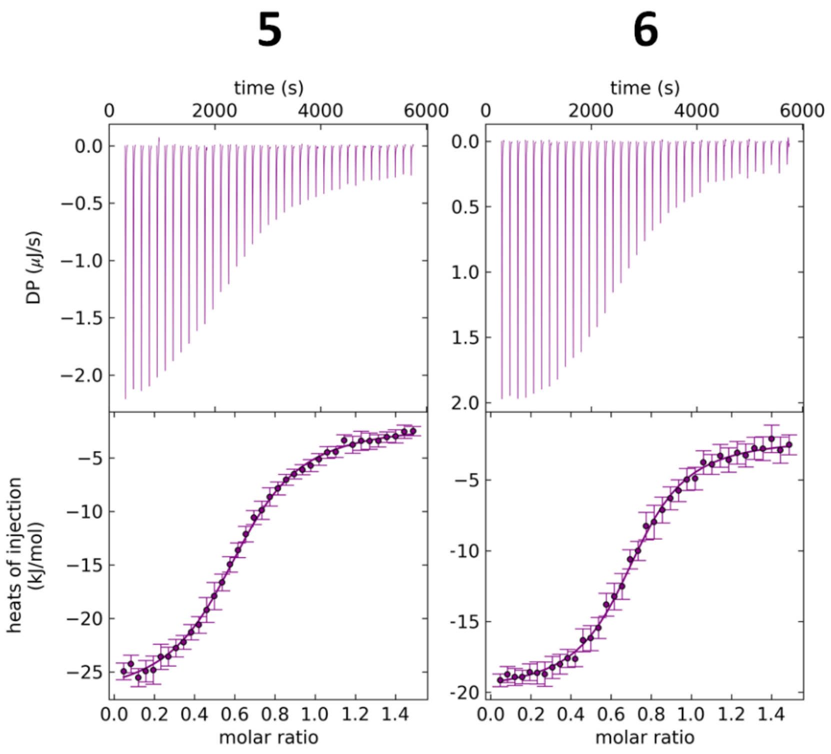

Figure S2: Examples of raw thermograms and integrated heat values for one of at least three displacement ITC measurements of the inhibitors **5** – **6**. For the thermogram on the top the y-axis shows the differential power in µJ/s and the x-axis the measuring time in s. The y-axis of the evaluated data below shows the heats of injections in kJ/mol and the x-axis the molar ratio.

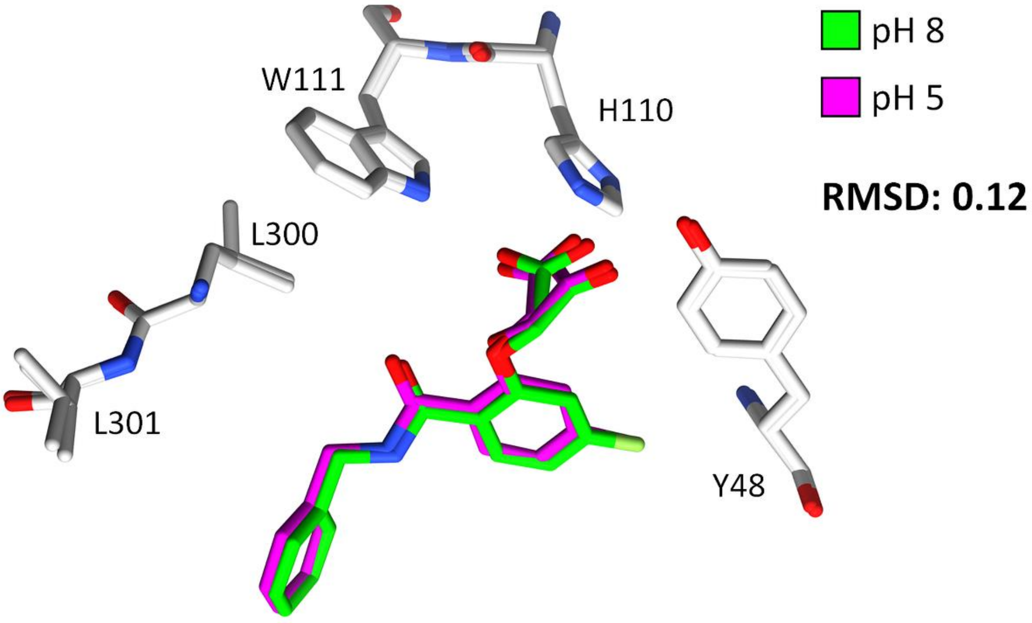

Figure S3: Superposition of the ligand geometries of **1** in the crystal structures with ALR-2 obtained by soaking at pH 5 (carbon atoms: magenta) and pH8 (carbon atoms: green). Selected residues of the active site are also shown (carbon atoms: white).

Crystallographic Tables

Table S1: X-ray data collection and refinement statistics of inhibitors **3** – **5** in complex with ALR-2 wildtype.

	**3** (6TUF)	**4** (6TUC)	**5** (not deposited)
(A) Data collection and processing			
Beamline	Bessy 14.2	Bessy 14.2	Bessy 14.1
Wavelength [Å]	0.9184	0.9184	0.9184
Space group	$P12_11$	$P12_11$	$P12_11$
Unit Cell parameters:			
a, *b*, *c* [Å]	47.2, 66.4, 49.2	47.2, 66.5, 49.2	47.3, 66.7, 49.4
α, *β*, *γ* [°]	90.0, 92.4, 90.0	90.0, 92.1, 90.0	90.0, 92.8, 90.0
Matthews coef. [$Å^3 \cdot Da^{-1}$]	2.2	2.1	2.2
Solvent content [%]	43	43	43
(B) Diffraction Data[a]			
Resolution range [Å]	47.13 – 1.15 (1.22 – 1.15)	49.19 – 1.06 (1.12 – 1.06)	39.68 – 0.93 (099 – 0.93)
Unique reflections	105125 (16246)	132582 (20046)	198129 (28823)
$R(I)_{sym}$ [%][b]	4.0 (50.7)	7.6 (37.1)	4.2 (30.6)
Completeness [%]	97.3 (93.3)	96.3 (90.3)	96.5 (87.0)
Redundancy	3.7 (3.5)	3.6 (3.4)	3.4 (2.9)
$I/\sigma\ (I)$	19.2 (2.3)	14.8 (3.6)	15.0 (2.8)
(C) Refinement			
Resolution range [Å]	34.83 – 1.15	49.24 – 1.06	38.61 – 0.93
Reflections used in refinement			
work	99878	125952	188222
free	5257	6630	9907
Final R values			
work [%][c]	12.9	12.6	10.4
free [%][d]	15.2	14.3	11.7
Number of protein residues	316	313	316
$NADP^+$ atoms	48	48	48
Inhibitor atoms	40	30	17
Water molecules	375	463	454
Other inhibitor atoms	13	13	13
RMSD bonds			
Bond length [Å]	0.006	0.006	0.006
Bond angles [°]	0.99	1.00	1.04
Ramachandran plot[e]			
favored regions [%]	91.0	89.5	91.4
additional allowed reg. [%]	9.0	10.5	8.6
generously allowed reg. [%]	0.0	0.0	0.0
Mean *B*-Factor [$Å^2$][f]			
Protein	13.4	8.8	10.1
Inhibitor	18.1	15.0	23.3
Water molecules	25.9	21.7	24.0
$NADP^+$	8.8	4.7	6.5
Other inhibitors	14.8	8.2	10.7

[a] values in parenthesis are statistics for the highest resolution shell. [b] $R(I)_{sym} = \frac{\Sigma|I - \langle I \rangle|}{\Sigma|I|} \cdot 100$ for which *I* = observed intensity and <*I*> = statistically weighted average intensity of multiple observations. [c] Calculated by MOLEMAN.[1] [d] R_{free} = same definition as for R_{work} for a cross validation set of ≈ 5% of the reflections. [e] Calculated by PROCHECK.[2] [f] $R_{work} = \frac{\Sigma|F_O - F_C|}{\Sigma|F_O|} \cdot 100$ for which F_O = observed structure factor amplitudes and F_C = calculated structure factor amplitudes.

Table S2: X-ray data collection and refinement statistics of inhibitor **6** in complex with ALR-2 wildtype

	**6** (6SYW)
(A) Data collection and processing	
Beamline	Bessy 14.1
Wavelength [Å]	0.91841
Space group	$P12_11$
Unit Cell parameters:	
a, *b*, *c* [Å]	47.3, 66.9, 49.3
α, *β*, *γ* [°]	90.0, 92.0, 90.0
Matthews coef. [$Å^3 \cdot Da^{-1}$]	2.2
Solvent content [%]	43
(B) Diffraction Data[a]	
Resolution range [Å]	47.29 – 0.93 (0.99 – 0.93)
Unique reflections	181142 (19356)
$R(I)_{sym}$ [%][b]	5.0 (45.8)
Completeness [%]	88.1 (58.3)
Redundancy	4.5 (3.8)
$I/\sigma\ (I)$	15.7 (2.5)
(C) Refinement	
Resolution range [Å]	39.69 – 0.93
Reflections used in refinement	
work	172084
free	9058
Final R values	
work [%][c]	10.9
free [%][d]	12.3
Number of protein residues	316
$NADP^+$ atoms	48
Inhibitor atoms	38
Water molecules	463
Other inhibitor atoms	13
RMSD bonds	
Bond length [Å]	0.010
Bond angles [°]	1.22
Ramachandran plot[e]	
favored regions [%]	89.9
additional allowed reg. [%]	10.1
generously allowed reg. [%]	0.0
Mean *B*-Factor [$Å^2$][f]	
Protein	9.9
Inhibitor	18.4
Water molecules	23.8
$NADP^+$	6.8
Other inhibitors	10.4

[a] values in parenthesis are statistics for the highest resolution shell. [b] $R(I)_{sym} = \frac{\Sigma|I-<I>|}{\Sigma|I|} \cdot 100$ for which *I* = observed intensity and <*I*> = statistically weighted average intensity of multiple observations. [c] Calculated by MOLEMAN.[1] [d] R_{free} = same definition as for R_{work} for a cross validation set of ≈ 5% of the reflections. [e] Calculated by PROCHECK.[2] [f] $R_{work} = \frac{\Sigma|F_O-F_C|}{\Sigma|F_O|} \cdot 100$ for which F_O = observed structure factor amplitudes and F_C = calculated structure factor amplitudes.

Supplementary References

1. Kleywegt, G. J., Zou, J. Y., Kjeldgaard, M. & Jones, T. A. *International Tables for Crystallography Volume F: Crystallography of biological macromolecules*. (2001). doi:doi: 10.1107/97809553602060000106.

2. Laskowski, R. A., MacArthur, M. W. ., Moss, D. S. . & Thornton, J. M. M. J. PROCHECK: A program to check the stereochemical quality of protein structures. *J. Appl. Crystallogr.* **26**, 283–291, 1993.
